# Survival from bladder cancer in England and Wales up to 2001

**DOI:** 10.1038/sj.bjc.6604599

**Published:** 2008-09-23

**Authors:** A Shah, B Rachet, E Mitry, N Cooper, C M Brown, M P Coleman

**Affiliations:** 1 Cancer Research UK Cancer Survival Group, Non-Communicable Disease Epidemiology Unit, Department of Epidemiology and Population Health, London School of Hygiene and Tropical Medicine, Keppel Street, London WC1E 7HT, UK; 2 Département of Hépatogastroentérologie et Oncologie Digestive, Centre Hospitalo-Universitaire Ambroise-Paré, 9 avenue Charles de Gaulle, F-92100 Boulogne, France; 3 Social and Health Analysis and Reporting Division, Office for National Statistics (Room FG/114) 1 Myddelton Street, London EC1R 1UW, UK; 4 Medical Director, Eastern Cancer Registry, Addenbrooke's Hospital, Box 193 Oncology, Hills Road, Cambridge CB2 2QQ, UK

Approximately 9200 cancers of the urinary bladder are registered in England and Wales each year, of which 6500 in men (8% of all malignancies) and 2700 in women (3%) (Office for National Statistics, 2003). Bladder cancer ranks as the fourth most common in men and ninth in women, and it causes approximately 4300 deaths (2800 in men) a year in England and Wales.

Incidence increases with age, most steeply above the age of 60 years. The two-fold or greater excess in men is world-wide (Ferlay *et al*, 2004), and the excess is three-fold above the age of 60 years. Bladder cancer is uncommon below the age of 50 years, except in countries where it is linked with chronic schistosomal infestation of the bladder. Both incidence and mortality show deprivation gradients, with higher rates in more deprived areas, but the differences are small (Quinn *et al*, 2001).

Incidence rose steadily in both sexes during the 1970s and 1980s, reaching a peak of approximately 31 cases per 100 000 per year in men and 9 per 100 000 in women by 1990. Incidence has fallen by approximately 10% since then in both sexes. Annual death rates in men have fallen by a third since the early 1990s (from 12 to 8 per 100 000), but the death rate in women (3 per 100 000) has not changed (Quinn *et al*, 2001).

Most bladder cancers (90%) are urothelial (transitional cell) carcinomas, often arising on the surface of small papillary tumours. The remaining 10% comprise mainly squamous cell carcinomas and adenocarcinomas. European recommendations for coding bladder tumours changed in 1995 to exclude some urothelial papillary tumours of the bladder that would previously have been classified as invasive (Pheby *et al*, 1995). Similar recommendations were implemented by UK cancer registries, but only for tumours registered from 2000 (UK Association of Cancer Registries, 2004).

Tobacco smoking confers a two- to three-fold risk. Occupational exposure to aromatic amines and polycyclic aromatic hydrocarbons in the chemical, rubber, transport and dye industries can also cause bladder cancer. Chemotherapeutic drugs, such as cyclophosphamide, and therapeutic irradiation of the pelvic region also increase the risk (Silverman *et al*, 2006).

Haematuria or pain often leads to the diagnosis. Approximately 75% of tumours are superficial (confined to the bladder mucosa or submucosal layer without muscle invasion) and these can be treated by transurethral resection. Tumours that have invaded muscle may require open surgery and a combination of intravesical or systemic chemotherapy, immunotherapy and radiotherapy.

Approximately 12% (21 000) of all the bladder tumours registered in England and Wales during the period 1986–1999 were coded as *in situ* (behaviour code 2) or uncertain if benign or malignant (behaviour code 1). The proportion of tumours in these categories has risen very sharply, from virtually zero during 1986–1990 (Coleman *et al*, 1999) to approximately 23% during the 1990s (data not shown). Large regional variations were also seen. These tumours were considered *ab initio* as ineligible for inclusion in the survival analyses.

Of the 160 614 eligible patients with a tumour explicitly coded as a malignant, invasive (behaviour code 3), primary cancer of the bladder, some 5% were excluded because their recorded duration of survival was zero (date of diagnosis same as date of death). Some of these patients will have been diagnosed on the day of death, but in many cases the cancer registration was based solely on the death certificate; hence, the date of diagnosis was unknown. It was not possible to distinguish these cases reliably in the available data; hence, all patients with ‘zero survival’ were excluded. The proportion of patients excluded for this reason was the same for all deprivation groups (data not shown). Only patients for whom the bladder cancer was the first primary malignancy were retained in the analyses; 5% of patients known to have had a previous primary cancer at some time since 1971 were excluded. Finally, 1.6% of patients were excluded because their vital status was unknown on 5 November 2002, when the data were extracted for analysis. In all, more than 141 500 patients were included in the analyses (88% of those eligible).

## Survival trends

**Figure 1:**
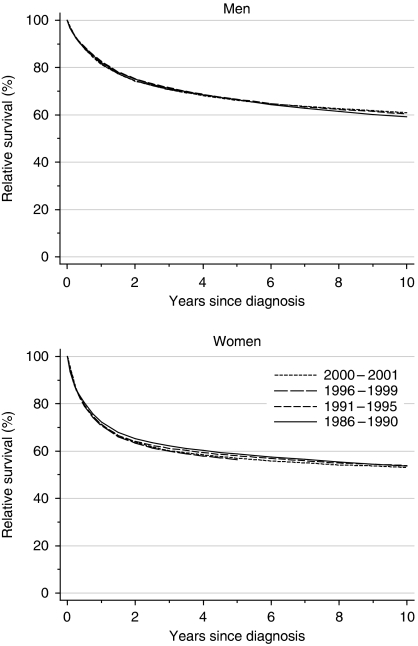
Relative survival (%) up to 10 years after diagnosis by sex and calendar period of diagnosis: England and Wales, adults (15–99 years) diagnosed during 1986–1999 and followed up to 2001. Survival estimated with cohort or complete approach (1986–1990, 1991–1995, 1996–1999) or hybrid approach (2000–2001) (see Rachet *et al*, 2008).

For bladder cancer patients diagnosed during 1996–1999, relative survival at 1 year was 82 and 71% for men and women, respectively, and 67 and 56% at 5 years (Table 1, Figure 1). Survival barely changed at all between the late 1980s and the late 1990s: deprivation-adjusted trends were very close to zero. Ten-year survival for patients diagnosed in the early 1990s was 60% for men and 54% for women.

The most striking feature is the persistent 6–10% survival advantage for men at 1, 5 and 10 years after diagnosis.

Examination of patients’ survival experience during 2000–2001 with hybrid analysis (Brenner and Rachet, 2004) does not suggest that survival up to 10 years after diagnosis is likely to increase in the near future (Table 1).

## Deprivation

**Figure 2:**
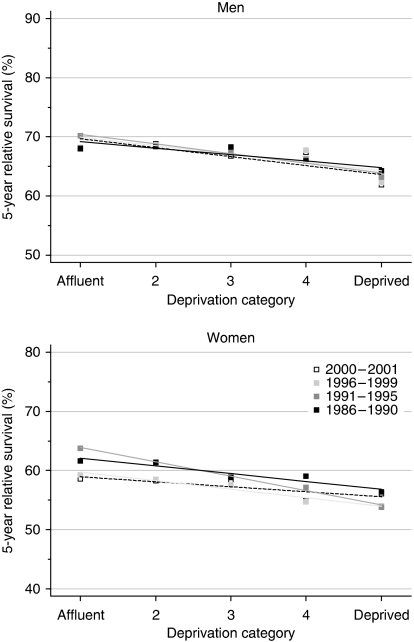
Trends in the deprivation gap in 5-year relative survival (%) by sex and calendar period of diagnosis: England and Wales, adults (15–99 years) diagnosed during 1986–1999 and followed up to 2001.

The deprivation gap in survival at 1, 5 and 10 years between the most affluent and the most deprived groups ranged from −4 to −10%. This was remarkably consistent for both men and women diagnosed from the late 1980s to the late 1990s. All the estimates of the deprivation gap in survival were statistically significant at the 1% level (Table 2, Figure 2).

The deprivation gap in survival up to 10 years widened slightly for both sexes, but the rate of change every 5 years was not itself statistically significant.

Hybrid analysis does not suggest any imminent change in the deprivation gap in survival up to 10 years for men, although some reduction of the socioeconomic differences may occur for women (Table 2).

## Comment

Survival for bladder cancer patients diagnosed in England and Wales during 1996–1999 seems to have been no higher than for patients diagnosed during 1986–1990, a decade earlier. Since survival up to 5 years did not increase by more than 2–3% between the early 1980s and the late 1980s either (Coleman *et al*, 1999), it would seem that bladder cancer survival in England and Wales as a whole has not increased much for almost 20 years.

The large and significant deprivation gap in survival between the most affluent and deprived patients (−4 to −10%) has not shrunk either.

The male survival advantage for bladder cancer (6–10%) is larger than for cancers of the upper urinary tract (2–4%). This has been a consistent feature for both cancers since the 1970s (Coleman *et al*, 1999). It is all the more striking because among the other common cancers, only breast cancer shows a survival advantage for men.

Changes in the spectrum of what is recorded – and treated – as an invasive malignant tumour can affect the interpretation of time trends in population-based cancer incidence and survival. Trends are more difficult to interpret for bladder cancer than for many other cancers, because of difficulties in the pathological definition of disease, variation between cancer registries in coding practice, and changes over time in both factors. Thus, the proportion of bladder tumours coded as *in situ* or of uncertain behaviour in England and Wales jumped from virtually zero for 1986–1990 to an average of 23% for 1996–1999. Similarly, the proportion of *in situ* tumours rose from 1 to 7% in the United States during the 1970s and has continued to rise since then (Silverman *et al*, 2006). Small biopsy fragments taken at cystoscopy may not enable the pathologist to determine unequivocally if the tumour is invasive or *in situ*, but the speed and magnitude of this shift must reflect changes in the pathological classification and registry coding of bladder tumours more than true change in the pathology of disease.

The declining incidence of invasive cancer of the bladder probably reflects earlier declines in tobacco smoking, but it may also echo this shift in the recorded spectrum of malignant behaviour. An increasing proportion of bladder tumours is now being coded as *in situ* or uncertain if benign or malignant. Some of these tumours are of low invasive potential (Newling *et al*, 1995). The recommended change in coding practice by European registries was made in 1995, but pathological practice may have changed before that. If a substantial proportion of bladder tumours that were classified by the pathologist (and coded by the cancer registry) as invasive during the 1980s would have been coded as *in situ* or of uncertain malignancy in the late 1990s, this would tend to cause both a decline in the recorded incidence of invasive cancer of the bladder and – as those low-grade tumours would also be excluded from survival analysis – a decline in survival from bladder cancer.

Regional variation within England and Wales in the percentage of bladder tumours coded as *in situ* or of uncertain malignancy is very wide. The overall percentage of *in situ* tumours during 1986–1999 ranged from less than 1 up to 27% among the 10 cancer registries (national average 8.1%). In some registries, it rose from less than 1% in 1986–1990 to 35% by 1996–1999, whereas in others it was stable at 1–2% (data not shown). A similar pattern was seen for tumours of benign or uncertain behaviour, the percentage ranging from 0.4 to 31% of all bladder tumour registrations during the period 1986–1999 (national average 3.4%), with similarly disparate trends among registries over that period. This must overstate any true underlying regional variation, and it reflects differences in pathological classification and registry coding practice. The relevance of these patterns is that the national data for the period 1986–1999 do not comprise a consistently defined group of bladder cancers.

Thus, a study of 1500 bladder cancer patients diagnosed in the West Midlands in 1990–1991 (Begum *et al*, 2004) found a socioeconomic gradient in crude (all-cause) survival, but not in cause-specific survival (deaths certified as due to bladder cancer), which should be very close to relative survival if death certification is accurate. The difference in crude survival at 5 years between the most affluent and most deprived groups (7%) was similar to that seen for West Midlands and for England as a whole during 1986–1990 (Coleman *et al*, 1999), but the survival estimates themselves (56–63%) were some 15% higher. These discrepancies suggest both errors in attribution of the cause of death and/or selection bias: fewer tumours were classified as *in situ* or benign in the West Midlands during 1986–1999 than in other regions of England (data not shown).

Bladder cancer survival rates observed in the late 1990s may therefore reflect a more aggressive spectrum of bladder tumours than those retained in survival analyses for the 1980s. This might help to explain why survival from bladder cancer seems to have improved so little between the 1980s and the 1990s.

More robustly interpretable information on population-based bladder cancer survival trends will require greater consistency in pathological definition and coding than seems to be available at present; this will take some time. In the meantime, examination of survival trends by stage of disease at diagnosis should prove informative, particularly if early-stage disease were excluded.

The shift since the mid-1990s towards a more malignant spectrum of bladder tumours that have been classified and coded as invasive in most registries may partly explain the overall lack of improvement in bladder cancer survival in England and Wales, but it does not explain why the wide socioeconomic inequalities in survival have not been reduced; nor does it explain the persistent sex difference in survival from bladder cancer.

## Figures and Tables

**Table 1:** Trends in relative survival (%) by sex, time since diagnosis and calendar period of diagnosis: England and Wales, adults (15–99 years) diagnosed during 1986–1999 and followed up to 2001

		**Calendar period of diagnosis^a^**						**Average change (%)**		**Prediction^c^ for patients**	
		**1986–1990**		**1991–1995**		**1996–1999**		**every 5 years^b^**		**diagnosed during 2000–2001**	
**Time since diagnosis**		**Survival (%)**	**95% CI**	**Survival (%)**	**95% CI**	**Survival (%)**	**95% CI**	**Survival (%)**	**95% CI**	**Survival (%)**	**95% CI**
1 year	Men	**82.2**	(81.7, 82.6)	**82.7**	(82.3, 83.1)	**81.6**	(81.1, 82.1)	**0.0**	(−0.9, 0.9)	**81.4**	(80.6, 82.1)
	Women	**72.2**	(71.4, 73.0)	**71.2**	(70.4, 72.0)	**70.6**	(69.7, 71.5)	**0.2**	(−1.5, 1.9)	**71.1**	(69.8, 72.4)
5 years	Men	**66.3**	(65.7, 67.0)	**66.6**	(66.0, 67.2)	**66.5**	(65.6, 67.4)	**0.7**	(−0.7, 2.2)	**66.1**	(65.1, 67.2)
	Women	**58.7**	(57.7, 59.8)	**58.0**	(57.1, 59.0)	**56.3**	(55.0, 57.6)	**−0.8**	(−3.0, 1.5)	**57.0**	(55.4, 58.6)
10 years	Men	**59.1**	(58.3, 59.9)	**60.3**	(59.4, 61.3)			**2.7**	(−0.7, 6.0)	**60.9**	(59.6, 62.2)
	Women	**53.6**	(52.4, 54.8)	**53.9**	(52.6, 55.2)			**3.6**	(−1.2, 8.3)	**53.0**	(51.2, 54.9)

CI=confidence interval.

a Survival estimated with cohort or complete approach (see Rachet *et al*, 2008).

b Mean absolute change (%) in survival every 5 years, adjusted for deprivation (see Rachet *et al*, 2008).

c Survival estimated with hybrid approach (see Rachet *et al*, 2008).

**Table 2:** Trends in the deprivation gap in relative survival (%) by sex, time since diagnosis and calendar period of diagnosis: England and Wales, adults (15–99 years) diagnosed during 1986–1999 and followed up to 2001

		**Calendar period of diagnosis^a^**						**Average change** (%)		**Prediction^c^ for patients**	
		**1986–1990**		**1991–1995**		**1996–1999**		**every 5 years^b^**		**diagnosed during 2000–2001**	
**Time since diagnosis**		**Deprivation gap (%)**	**95% CI**	**Deprivation gap (%)**	**95% CI**	**Deprivation gap (%)**	**95% CI**	**Deprivation gap (%)**	**95% CI**	**Deprivation gap (%)**	**95% CI**
1 year	Men	−4.1**	(−5.5, −2.8)	−5.3**	(−6.6, −4.1)	−4.6**	(−6.1, −3.1)	**−0.3**	(−1.4, 0.7)	−4.8**	(−6.9, −2.6)
	Women	−4.3**	(−6.8, −1.9)	−7.0**	(−9.4, −4.7)	−6.5**	(−9.2, −3.8)	**−1.2**	(−3.1, 0.7)	−5.3**	(−9.2, −1.5)
5 years	Men	−4.4**	(−6.3, −2.4)	−6.5**	(−8.4, −4.7)	−5.7**	(−8.2, −3.1)	**−0.9**	(−2.5, 0.8)	−6.1**	(−9.1, −3.0)
	Women	−5.3**	(−8.3, −2.3)	−9.7**	(−12.5, −6.8)	−5.8**	(−9.5, −2.0)	**−0.7**	(−3.2, 1.8)	**−3.4**	(−8.1, 1.4)
10 years	Men	−3.8**	(−6.2, −1.3)	−5.9**	(−8.8, −3.1)			**−2.2**	(−5.9, 1.5)	−6.5**	(−10.2, −2.8)
	Women	−6.0**	(−9.6, −2.5)	−10.2**	(−14.1, −6.2)			**−4.1**	(−9.4, 1.2)	**−2.5**	(−8.0, 3.1)

CI=confidence interval.

a Survival estimated with cohort or complete approach (see Rachet *et al*, 2008).

b Mean absolute change (%) in the deprivation gap in survival every 5 years, adjusted for the underlying trend in survival (see Rachet *et al*, 2008).

c Survival estimated with hybrid approach (see Rachet *et al*, 2008). ^**^P<0.01.
